# Researches on the Hydrides and Hydrates

**Published:** 1894-06-09

**Authors:** Benjamin Ward Richardson


### Researches on the Hydrides and Hydrates—*Continued.*

By Sir Benjamin Ward Richardson, M.D., F.R.S.

### HYDRIDE OF AMYL.

I dwelt in last paper on methyl hydride, a permanent gas, and I might pass to some details respecting the ethyl and butyl hydrides; but as they are also gases, and differ only from the methyl specimen in that they are heavier, I may pass them over with the remark that perhaps the butyl hydride might be more useful than the methyl because it could be condensed into a liquid with less pressure, and would be a rather more stable compound. I proceed now to the first number of the series of the hydrides that is presented naturally to us in the fluid state, namely, the hydride of amyl.

Hydride of amyl may be made by adding iodide of amyl to water with the addition of zinc finely powdered, and subjection to heat up to 288 deg. Fahr. The heat has to be sustained for several hours, and then distillation may commence. The product is a mixture of the substance known as amylene (which has been used as an anæsthetic) and the amyl hydride. The mixture is treated with caustic potash, and is then rectified from a water-bath at 95 deg. Fahr., precaution being taken not to allow the temperature to pass that point. The distillate is received in a flask surrounded by a freezing mixture, and is then treated with fuming sulphuric acid, which retains the amylene, and the hydride is distilled over. The process is known as Frankland's, but it is too elaborate for obtaining the hydride on a large scale for experimental purposes. Fortunately, the substance is found ready made in some of the petroleums, from which it can be obtained in abundant quantity. The commercial name of it is rhigolene. The specific gravity of this fluid is 0·625, and it boils at 86 deg. Fahr., 30 centigrade. It is a very mobile liquid, and must be retained with great care in well-stoppered bottles and in a cool place. Practically it is insoluble in water and in blood. It is colourless and inodorous, and, in form of vapour, can be breathed without creating irritation.

In the year 1867 some American chemists, whose names have escaped me, distilled from petroleum a fluid which, on being volatilised, produced a vapour that caused anæsthesia by inhalation; and it is a curious fact that an oil-tanked steamship named the *Kasbek*, while recently sailing from Batoum to London, gave off from her hold a vapour which partially narcotised some of the seamen, and which produced similar effects on the captain, who allowed himself to be exposed to it. The vapour given off was no doubt a mixture of the hydrides—hydrides of methyl, propyl, butyl, and amyl. The fact observed in 1867 led me to investigate more carefully the amyl hydride to determine its anæsthetic power. I subjected it to experiment, and found that it might be applied as a general anæsthetic. Forty per cent. of the vapour of this hydride, diffused through air, acted very rapidly; but owing to its extreme volatility the administration had to be kept up almost without cessation. The effects produced were extremely rapid, narcotism being established in from forty to eighty seconds; but as the recovery from it was rapid—commencing in a very few seconds—none but short operations, such as the extraction of a tooth, could be carried out under its action.

In order to put the vapour to its full test, as an anæsthetic, I subjected myself to it by breathing it from a vulcanite inhaler. I found it very pleasant to breathe; it caused no cough, no irritation, no symptom except a pleasant sensation of glow or warmth in the chest. After six inspirations I experienced changes in the cerebral circulation; giddiness, and inability to stand, with the swaying movement, or sense of movement, which commonly marks the first degree of anæsthetic sleep. In a little time I lost consciousness, as I learned afterwards from those with me, but the inhaler being removed, I quickly recovered, and in three minutes felt perfectly well. I experienced later on neither nausea, headache, nor chilliness, nor did I, through any of the period, experience the sensations or dreams that are brought about by nitrous oxide inhalation. I had no sense of exhilaration, such as nitrous oxide excites; the action, in fact, was entirely negative, except for the slight warm glow which was felt in the chest.

I noticed afterwards, in administering the vapour to the lower animals—to the extent of inducing deep insensibility—that the insensibility was usually attended by slight convulsive movements. I observed also that there was tendency to reduction of temperature of the body under its use, but only in a very moderate degree, a fourth of Fahrenheit's scale; but when the animals were allowed to sleep to death the temperature fell before actual death one and a-half degrees Fahrenheit. In this extreme state the pupil dilated, and immediately after death the heart, well charged with blood on both sides, held blood of a dark colour on the left side as well as on the right; coagulation was not interfered with, and the corpuscles were uninjured. The lungs were charged with blood, as is common when both sides of the heart contain blood, and they were natural in colour. The brain presented no sign of congestion or injury, and muscular irritability was long retained.

For two reasons I did not press the administration of amyl hydride for anæsthesia; first, because it did not seem to me to offer any advantages over other anæsthetics; and, secondly, because in the inferior animals I twice witnessed sudden collapse and death during its administration. and found, at the post-mortem, the free vapour, in bubbles, in the blood. But I used it with great effect in restoring to activity frozen parts that had been rendered insensible by cold; dead parts, and even animals, batrachians, that were apparently altogether dead from cold.

One of the most useful applications of the hydride of amyl consists in employing it in the form of spray in order to produce local insensibility. Sprayed on a living part it causes almost immediate congelation and sufficient local anæsthesia for the performance of small operations. For short operations it answers in this way well, and as we can follow the knife of the surgeon with it without injury, it suffices even for some deep operations and for some prolonged ones when they are superficial. I combined it with anhydrous ether in the fluid known as "Anæsthetic mixture for local anæsthesia." This mixture is composed of equal parts of ether and the hydride.

